# Chromatic Mechanical Response in 2-D Layered Transition Metal Dichalcogenide (TMDs) based Nanocomposites

**DOI:** 10.1038/srep34831

**Published:** 2016-10-07

**Authors:** Vahid Rahneshin, Farhad Khosravi, Dominika A. Ziolkowska, Jacek B. Jasinski, Balaji Panchapakesan

**Affiliations:** 1 Small Systems Laboratory, Department of Mechanical Engineering, Worcester Polytechnic Institute, Worcester, MA 01609, USA; 2 Conn Center for Renewable Energy Research, University of Louisville, Louisville, KY 40292, USA; 3 Faculty of Physics, University of Warsaw, Pasteura 5, 02-093 Warsaw, Poland

## Abstract

The ability to convert photons of different wavelengths directly into mechanical motion is of significant interest in many energy conversion and reconfigurable technologies. Here, using few layer 2H-MoS_2_ nanosheets, layer by layer process of nanocomposite fabrication, and strain engineering, we demonstrate a reversible and chromatic mechanical response in MoS_2_-nanocomposites between 405 nm to 808 nm with large stress release. The chromatic mechanical response originates from the *d* orbitals and is related to the strength of the direct exciton resonance A and B of the few layer 2H-MoS_2_ affecting optical absorption and subsequent mechanical response of the nanocomposite. Applying uniaxial tensile strains to the semiconducting few-layer 2H-MoS_2_ crystals in the nanocomposite resulted in spatially varying energy levels inside the nanocomposite that enhanced the broadband optical absorption up to 2.3 eV and subsequent mechanical response. The unique photomechanical response in 2H-MoS_2_ based nanocomposites is a result of the rich *d* electron physics not available to nanocomposites based on *sp* bonded graphene and carbon nanotubes, as well as nanocomposite based on metallic nanoparticles. The reversible strain dependent optical absorption suggest applications in broad range of energy conversion technologies that is not achievable using conventional thin film semiconductors.

The ability to convert photons into mechanical motion is of significant importance for many energy conversion technologies. Establishing an optical-mechanical interface has been attempted since 1881 when A.G. Bell used the optoacoustic effect (photophone) to produce sound in a gas with chopped beam of sun light^1^. However, only few materials exist that can convert photons of different wavelengths into mechanical motion that is large enough for practical import. Examples of materials that can directly convert light of different wavelengths into mechanical motion include range of organic photochromic compounds^2^,^3^, lead-lanthanum-zirconate-titanate (PLZT) ceramics^4^, carbon nanotube polymer composites with selective chirality distributions^5^ and more recently metallic nanoparticle-polymer composites^6^,^7^. Photomechanical actuators, motors, and micro-walking devices based on these materials have also been developed^2^,^3^,^4^,^5^,^6^. In general, most photomechanical actuators based on sp bonded carbon namely nanotube/graphene^8^,^9^,^10^,^11^,^12^ are triggered mainly using near infra-red light and they do not exhibit wavelength selectivity. The mechanism in these nanocomposites is non-radiative decay of photons resulting in localized thermal effect^8^,^9^,^10^,^11^,^12^. While, near infrared triggered mechanical response was recently reported in bulk MoS_2_ nanocomposites prepared using a shear mixing process, these nanocomposites neither exhibit wavelength selectivity nor enabled phase separated and layered nanocomposite structure which can bring the more interesting and unique structure based chromatic optical absorption in these materials^13^. While this field is growing rapidly with many applications reported from cantilever based photomechanical actuators^14^, photo-mechanical micro-grippers with milli-second (ms) time constants^15^, photo-thermal micro-pillar actuators^6^, micro-mirrors with large rotational angles^16^, nanopositioners^17^, and plastic motors^5^,^18^, there is still need for a material design that is simple, versatile, reversible, wavelength selective, scalable and encompasses large optical to mechanical stress response that is based on their unique structure based tunability in optical absorption at different wavelengths. Phase separated nanocomposites based on TMDs prepared using a layer by layer processing method may offer a new material design and approach for chromatic photomechanical actuation with large stress release owing to the van Hove singularities in the joint density of states in the visible region of the electromagnetic spectrum that could be useful in designing future wavelength selective reconfigurable technologies^19^.

Layered transition metal di chalcogenide (TMDs) provide intriguing opportunities to develop low cost, light and wavelength tunable stimuli responsive systems that are not possible with their conventional macroscopic counterparts. TMDs are stack of triple layers with transition metal layer between two chalcogen layers. While the atoms within the layers are chemically bonded using covalent bonds, the triple layers can be mechanically/chemically exfoliated due to weak van der Waals bonding between the layers. Due to the large optical absorption (~10^7^m^−1 ^19) in these materials, they are already being exploited for photocatalytic^20^, photoluminescence^21^, photo-transistors^22^ and solar cell^23^ applications. Bulk form of MoS_2_ has an indirect band gap of ~1.29 eV^24^ and single layer has a band gap of 1.9 eV^24^. The in-plane structure of MoS_2_ is determined by strong covalent bonds resulting from the overlap between the 4d and 3p electron orbitals of Mo and S respectively^25^. The elastic modulus and the breaking strength of an ideal defect-free single-layer MoS_2_ is expected to be E^2D^/9, where E^2D^ is the in-plane stiffness as per the theory of rupture and flow in solids^25^,^26^. The ultra-high strength is generated from the p orbitals of the chalcogen atoms which generate the σ bonds^19^. The large breaking strength (capable of sustaining elastic deformation of 25% before failure)^27^ together with large band gap and strong light-matter interaction in these materials has resulted in plethora of investigation on electronic, optical and magnetic properties of such layered ultra-thin semiconductors^27^,^28^,^29^,^30^,^31^,^32^,^33^.

Utilizing exfoliated MoS_2_, unique layer by layer process of nanocomposite fabrication, and strain engineering, here we present direct chromatic mechanical response in MoS_2_-polymer nanocomposites between 405 nm–808 nm. The advantage of our fabrication methods are as follows: (1) liquid phase exfoliation process resulting in high quality few layers with distinctive direct electron transition peaks, (2) phase separated nanocomposite due to the layer by layer processing, (3) access to the unique layer dependent optical absorption properties of MoS_2_ layers inside the nanocomposite, (4) the reported architectures enables the creation of spatially varying bandgap profiles of 2-D nanomaterials and study of resonance Raman scattering as a function of number of layers and (5) the design enables scalable and flexible process for developing stimuli responsive and energy conversion devices at macro, micro and nanoscopic length scales utilizing different polymeric systems. Further, the layer by layer process as reported here enables preservation of the intrinsic properties and eliminates environmental degradation effects such as oxidation. The reported photomechanical actuators based on this process not only showed strong and wavelength dependent light matter interaction in the visible region of the electromagnetic spectrum, but also showed unexpected deviations. Not only, we found that the photo-thermal energy transduction from the MoS_2_ crystals to the polymer chains is high resulting in high amplitudes of photomechanical actuation, it is also dependent on the wavelength of light and the photo-thermal actuation is better than graphene^17^ and carbon nanotubes^9^, which is unexpected given the low thermal conductivity of the MoS_2_ additives (52 W/m-K for few-layer MoS_2_^34^ versus 313 W/m-K for graphene thin films^35^).

## Results

**Figure 1:**
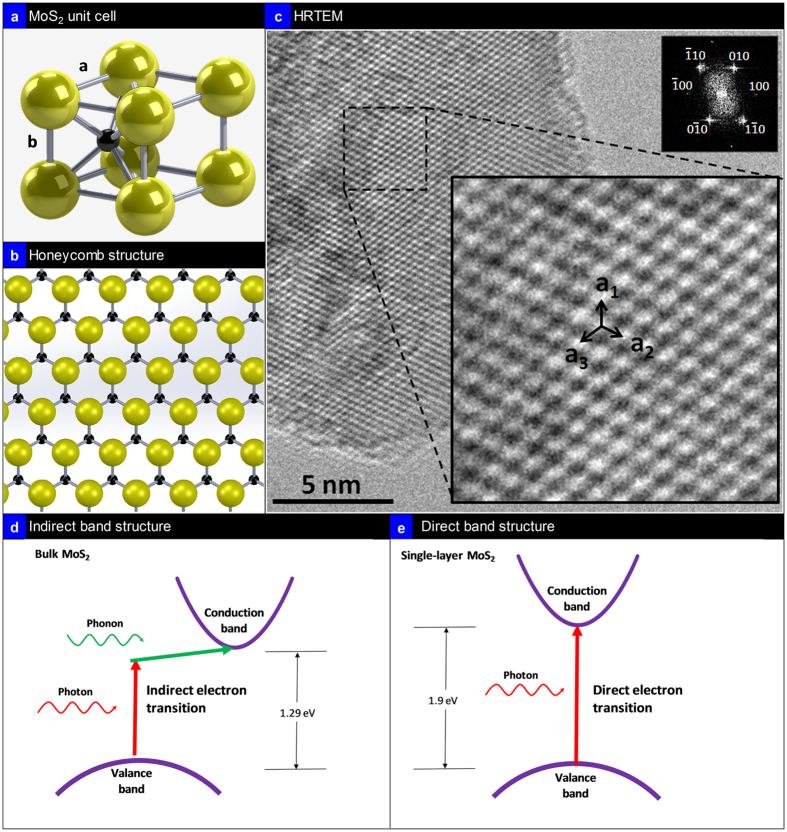
Crystal Structure. (**a**) Crystal structure of MoS_2_: S atoms in gold and Mo in black with unit cell parameter a and b, (**b**) top view of a single-layer MoS_2_ structure in honeycomb shape; (**c**) HRTEM of 2H-MoS_2_ (top insert is the Fast Fourier Transform (FFT) image showing the planes; slight distortion is due to the tilting of the flake in the TEM; a_1_, a_2_ and a_3_ presented HRTEM image is the Mo-Mo interatomic distance of 2.8 Å for 2H-MoS_2_ (**d–e**) schematic of the indirect electron transition in bulk MoS_2_ and direct electron transition in single layer MoS_2_ respectively.

Figure 1(a) presents the lattice schematic and Fig. 1(b) honeycomb structure of the semiconducting phase of 2H-MoS_2_. In the 2H-MoS_2_ lattice, each Mo atom is located at the center of a trigonal prism created by six S atoms. The lattice constant of 2H-MoS_2_ was reported to be: *a* = *b* = 3.14 Å^36^ and *c* = 12.3 Å^37^. A single layer of 2H-MoS_2_ is ~0.65 nm thick and can be exfoliated using scotch tape^38^ and liquid phase exfoliation techniques^39^. The High Resolution Transmission Electron Micrograph (HRTEM) of the MoS_2_ structure is presented in Fig. 1(c) with atoms of S and Mo indicated also showing the honey comb structure in (Fig. 1b). The distances a_1_, a_2_ and a_3_ measured from this image are close to the 2.8 Å value of the Mo–Mo interatomic distances in 2H-MoS_2_^40^. The insert in Fig. 1(c) presents the Fast Fourier Transform (FFT) of the HRTEM image indexing the crystallographic planes of the single layer 2H-MoS_2_. Small distortions and apparent non-uniform intensity distribution of the spots in FFT are due to a slight tilt of the flake with respect to the electron beam. The bulk form of MoS_2_ with an indirect band gap of ~1.29 eV^24^ (Fig. 1d), the photon absorption process is mainly dominated by electronic polarization and as a result, no wavelength selective optical absorption is observed, since no significant electronic transition happens from the valance to the conduction band. However, as the layers decrease in MoS_2_, the indirect band gap becomes so large that the material changes into a direct band gap semiconductor with Eg~1.9 eV(Fig. 1e)^24^ with intrinsic photoluminescence^21^. The low energy electronic states are dominated by 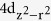, 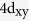, and 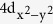 orbitals of Mo atoms^41^. MoS_2_ few layers are also known to have significant quantum yield (10,000 folds increase in luminescence) compared to bulk^24^. MoS_2_ show strong peak and enhanced light-matter interaction in the visible region of the electromagnetic spectrum due to the van Hove singularities in the density of states^19^.

**Figure 2:**
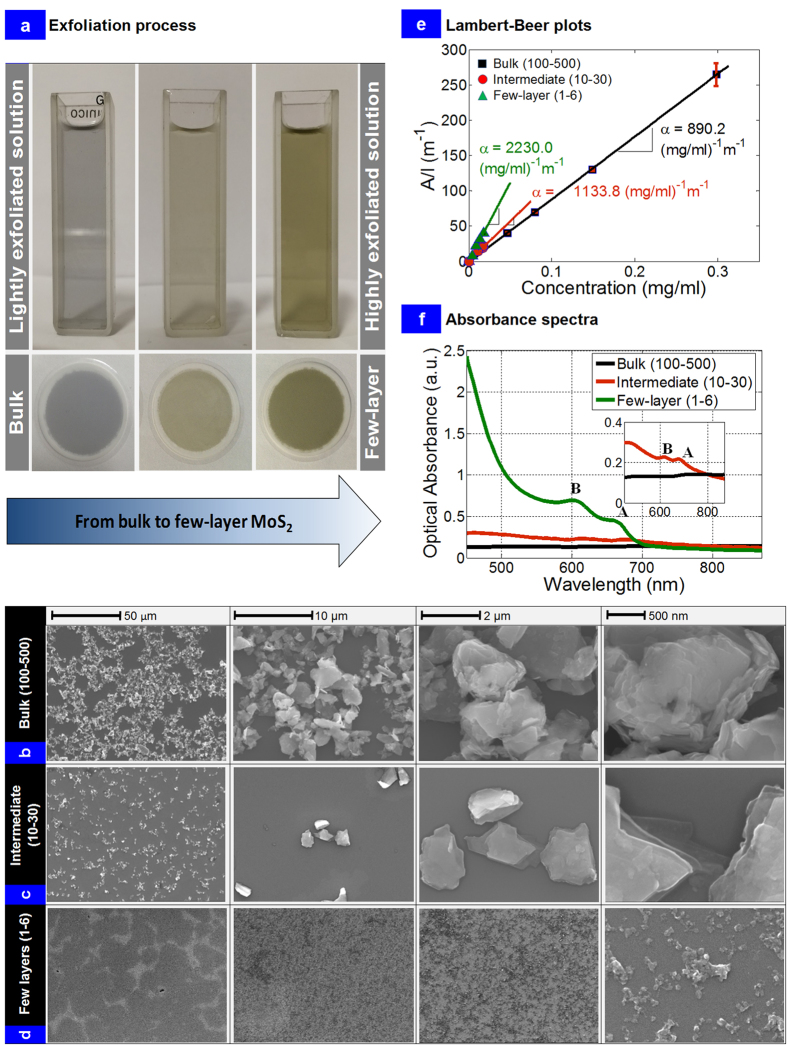
Liquid Phase Exfoliation and Characterization. (**a**) Suspensions after liquid phase exfoliation in bath sonicator followed by centrifugation and separation of layers into 3 distinct entities namely bulk (left: 100–500 layers), intermediate (middle: 10–30 layers) and few layers (right: 1–6 layers); bottom row corresponds to the bulk, intermediate and few layer films formed on an anodisc membrane from the exfoliated suspensions (**b–d**) SEM images of the three different suspensions showing the different morphology of the samples. The bulk were aggregates with particle size greater than 1μm, the intermediate samples were crystals with layered architecture with particle size ~1μm and the few layer samples with layered crystals ~100 nm in particle size; (**e**) Lambert-Beer plots characterized by *A/l = αC* provides the extinction coefficient for all the three types of samples; (**f**) UV-Visible spectroscopy of the bulk, intermediate and few layer samples in solution. The peaks marked A and B correspond to the direct exciton transition peaks at the “*K*” point.

The starting point for our photomechanical actuators is the liquid phase exfoliation of MoS_2_^39^ powders in ethanol followed by centrifugation and separation of the layers as presented in Fig. 2(a). Three types of samples were prepared namely (a) bulk: sonicated for 7 hours and no centrifugation (b) intermediate: sonicated for 50 hours and centrifuged for 45–120 minutes and layers separated (c) few layers: well characterized commercially available few layers in ethanol was purchased. The samples were characterized for number of layers using an SEM (Fig. 2(b–d)) and an AFM (Figure S1 Suppl.). It is observed that bulk samples had morphology of flakes (Fig. 2b), while the intermediate was well separated crystals. The crystals were also large measuring almost 1 μm in size (Fig. 2c) and the few layers were small in size measuring 100 nm in diameter (Fig. 2d). Further characterization for number of layers was done using an AFM (Figure S1 Suppl.). Three different regions were identified based on the Z-axis height measurements of the flakes using an AFM. Based on the number of layers, the suspensions were classified as bulk (100–500 layers), intermediate (10–30 layers) and few layers (1–6 layers) and henceforth the films and the nanocomposites made using these suspensions will be addressed as such throughout the paper. These suspensions were then filtered using vacuum filtration using an Anodisc membrane to yield MoS_2_ thin films (Fig. 2a, bottom row), with the same mass per area of the membrane (please see materials and methods for sample preparation). It can be observed the thin film samples on membrane have characteristic colors namely few layer films were deep green (1–6 layers), intermediate films were light green (10–30 layers) and bulk samples were grey (100–500 layers) as presented (Fig. 2a, bottom row). This suggests that the films themselves are capable of acting as optical filters at these low weight fractions.

Figure 2(e) presents the optical absorption spectroscopy of bulk, intermediate and few layer solutions using Lambert-Beer’s law characterized by *A/l* = α*C*, where *A/l* is the absorbance per length, α is the extinction coefficient, and *C* is the concentration. The *A/l* scaled linearly with *C* provided the values for the three types of solution. The calculated extinction coefficient for bulk α_bulk_ = 890.2 (mg/ml)^−1^ m^−1^; for intermediate layer α_intermediate-layer_ = 1133.8 (mg/ml)^−1^ m^−1^; and α_few-layer_ = 2230 (mg/ml)^−1^ m^−1^. The extinction coefficient increases with decrease in number of layers. Since extinction is a measure of absorption and scattering, and since the few layer nanoparticles were small (<100 nm), the large values of extinction is a result of optical absorption in the semiconducting few layers. Figure 2(f) presents the absorbance versus the wavelength of the three different solutions. It is seen that absorbance is high (25 times higher than bulk) for the few layer solution. The two peaks marked A (1.9 eV) and B (2.1 eV) correspond to the direct exciton transition at the *K* point^42^. The peaks are assigned to excitons involving the conduction band and the two valence bands (which are split due to spin orbit coupling) near the *K* point^42^. The absorbance of the intermediate and the bulk solution was small. The insert in Fig. 2(e) presents the magnified absorbance of the intermediate and the bulk solutions. The A and B peaks are also evident in the intermediate solution. The bulk solution showed no such peaks (suggesting no direct electron transition) with negligible absorption. The negligible absorption is a result of the high transmittance of light through the sample and geometric scattering of light due to the particle size of the bulk (>1 μm) and intermediate MoS_2_ (~1 μm) samples in solution.

**Figure 3:**
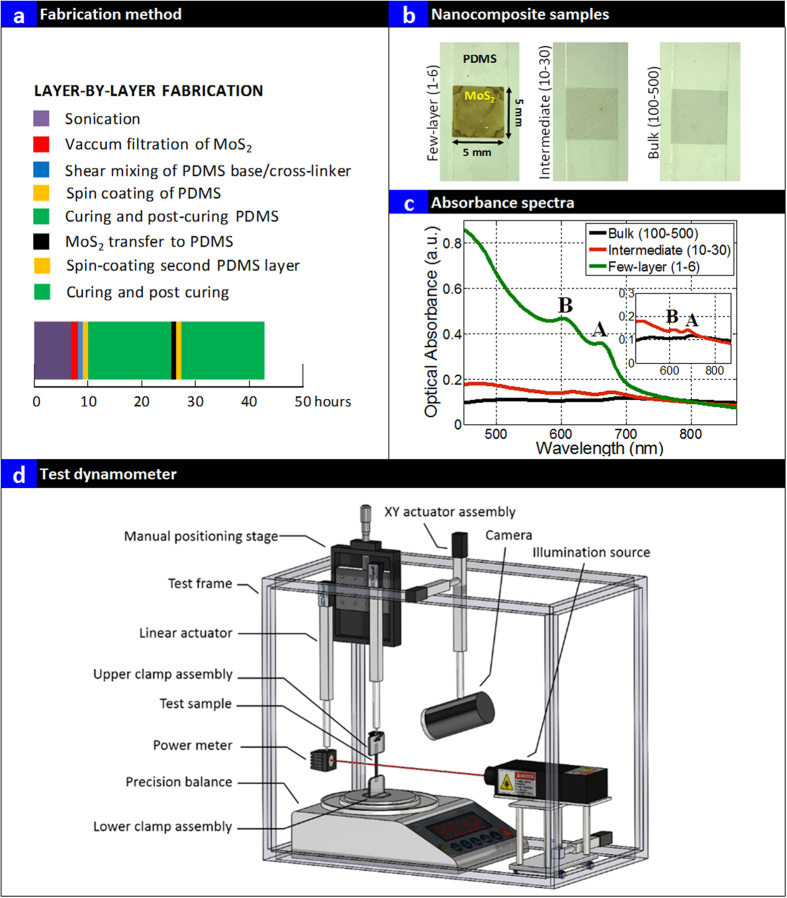
Layer by Layer Assembly of Nanocomposites and Testing. (**a**) Layer by Layer Fabrication Process; (**b**) Final nanocomposites formed using layer by layer process; (**c**) UV-Visible spectroscopy of the bulk, intermediate and few layer nanocomposites; insert is the magnified image of the optical absorbance of bulk and intermediate nanocomposites (**d**) Schematic of the photomechanical test assembly.

Figure 3(a) presents the nanocomposites prepared using layer by layer (LBL) process consisting of steps namely: sonication, vacuum filtration of exfoliated MoS_2_ suspensions, shear mixing of PDMS base with cross-linkers, formation of layer of cross-linked poly dimethyl siloxane (PDMS) on glass substrate using spin coating, curing and post-curing of PDMS, MoS_2_ transfer to the PDMS and spin coating another identical second layer of PDMS on to the transferred MoS_2_ layer and crosslinking and final step of curing and post-cure relaxation for 12 hours. The MoS_2_ concentrations for all the three samples were maintained at 0.1 wt. % and the final nanocomposites are presented in Fig. 3(b). The top and bottom PDMS layer were identical in weight and thickness. All the samples were identical in mass. Following preparation, the samples were cut into 50 mm × 5 mm strips for testing (Fig. 3(b)). Figure 3(c) presents the optical absorbance of the three types of nanocomposites measured using UV-Visible spectroscopy. The optical absorbance of the nanocomposites were similar to that of the solution samples as presented in Fig. 2(f) except the normalized absorbance of the Y-axis was lower. The absorbance of neat PDMS was measured and subtracted from the presented data on the nanocomposites. The few layer based nanocomposites showed the strongest optical absorbance (300 nm to 750 nm) compared to their intermediate and bulk counterparts. The characteristic exciton resonance peak A (~1.9 eV) and B (~2.1 eV) is seen suggesting existence of a direct bandgap inside the nanocomposite. Again, the absorbance of the intermediate and the bulk nanocomposites was small similar to the solution based samples. This suggest high transmittance of light through the sample and geometric scattering due to particle size that caused the lowered optical absorbance in intermediate and bulk nanocomposites at these low weight fractions. It should be noted that the absorbance was exactly the same at higher wavelengths (>800 nm, below the bandgap of the few layer samples) for all the three nanocomposite samples suggesting same weight fractions of the MoS_2_ additives and similar levels of scattering in all the nanocomposites^39^. The insert in Fig. 3(c) presents the magnified absorbance of the intermediate and the bulk nanocomposites. The A and B peaks are also evident in the intermediate nanocomposite. The bulk nanocomposite showed no characteristic A and B peaks with negligible absorption (suggesting no direct electron transition in bulk). Since the extinction of the few-layer samples was observed to be high, this suggest strong absorption of light in these nanocomposites (300 nm to 750 nm). Cross-sectional SEM images of the bulk nanocomposites (Figure S2, suppl.) suggest the two layers of PDMS with a layer of MoS_2_ in between. The particles were seen to be along the line indicated in red (Figure S2, Suppl.) with particle size about 1 μm. These investigations suggest most of the light is transmitted and geometric scattering due to particle size affect optical absorption in bulk nanocomposites. Photomechanical testing was done in an automated test system (Fig. 3d) and the details of the testing is reported elsewhere^12^.

**Figure 4:**
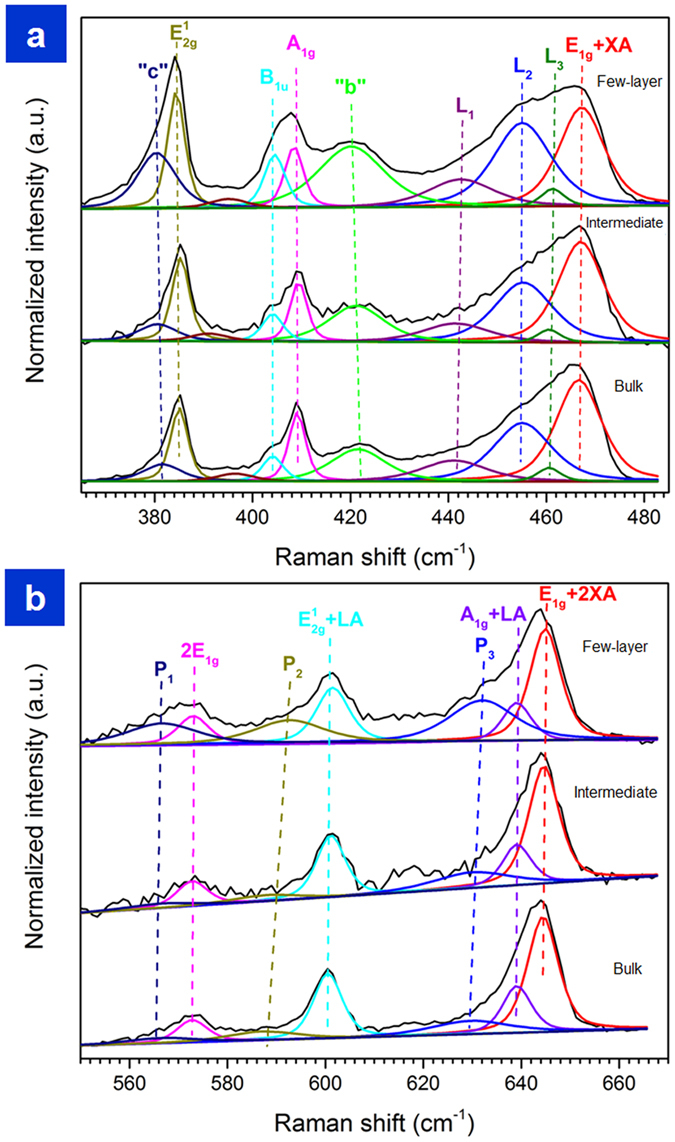
Raman Scattering. Resonant Raman Spectroscopy of bulk, intermediate and few layer nanocomposites. Presence of van Hove singularities “L_2_” suggest high optical absorption.

In order to better understand the internal lattice dynamics in bulk, intermediate and few layer nanocomposites, we performed resonant Raman scattering studies of our nanocomposites at room temperature as presented in Fig. 4. This is the first such measurement performed directly on the nanocomposite, which enables us to non-destructively understand the internal dynamics between bulk, intermediate and few layer samples.

Significant changes in the shape of lines are observed between bulk, intermediate and few layer samples in Fig. 4 (a,b). While the first order E^1^_2g_ (~384 cm^−1^) and A_1g_ (~409 cm^−1^) peaks, which correspond to the in-plane and out-of-plane vibrational modes of the S atoms with respect to the Mo atoms, are dominant features of the non-resonant Raman spectrum of 2H-MoS_2_^43^, these peaks are accompanied by a number of intense second-order Raman scattering process peaks enhanced by strong electron-phonon interactions in the resonant spectrum (Fig. 4a).

In the bulk nanocomposite, the strongest spectral feature is a broad line located around 460 cm^−1^ that consists of E_1g_ + XA (~466 cm^−1^) peak (XA is a transverse phonon from the vicinity of M point of the Brillouin zone)^44^,^45^ as well as of L_1_ (~441 cm^−1^) peak assigned to a second-order band (A_1g_(Γ) + E^2^_2g_(Γ)), L_2_ (~455 cm^−1^) peak to a van Hove singularity between K and M and L_3_ (~460 cm^−1^) peak due to LA′ phonon from the vicinity of M point^46^. Thus in bulk, the existence of van Hove singularity is expected to increase optical absorption in the visible region of the electromagnetic spectrum with increase in MoS_2_ additives. Other significant lower-frequency lines are the “b” (~420 cm^−1^) peak due to a two-phonon Raman process involving the emission of a dispersive quasi-acoustic phonon and an optical E^2^_1u_ phonon along the c-axis, the B_1u_(Γ) (~404 cm^−1^) mode, which is the Davydov couple of the A_1g_ (Γ) mode and the “c” (~380 cm^−1^) peak due to a dispersionless E^2^_1u_ (Γ) phonon^47^. In addition, several lines are also observed in the higher-energy region between 550 cm^−1^ and 700 cm^−1^(Fig. 4(b)), including the E_1g_(M)+2XA (~644 cm^−1^) peak^44^, the A_1g_(M)+LA(M) (~466 cm^−1^) peak, E^1^_2g_(M) + LA(M) (~600 cm^−1^) peak and 2E_1g_(Γ) (~572 cm^−1^) peak.

Several significant changes of the spectrum are observed with the decrease in the number of layers, especially for the few layer nanocomposite. First, the “b” peak shows higher intensity which is an indicative of the phonon involved that has a wave vector along the c-axis. The “c” and B_1u_ (Γ) peaks are also seen to increase in intensity. Both these peaks are Raman-inactive and their appearance suggests symmetry breaking^47^. Since the PDMS spectra were subtracted from the presented measurements, the peaks are indicative of the arrangement and interaction of the MoS_2_ atoms between the layers arranged in the few layer sample.

The decrease in the number of layers of the MoS_2_ crystal leads also to significant increase of the L_1_ and L_2_ peaks (Fig. 4a) in the few layer nanocomposites suggesting increase in optical absorption. Furthermore, in the higher-energy region of this sample, broad peaks labeled in Fig. 4(b) as P_1_, P_2_ and P_3_, located at ~566 cm^−1^, ~592 cm^−1^and ~632 cm^−1^, respectively, are seen to appear. These peaks also change in intensity with decrease in number of layers. The bulk and intermediate seem to be of similar intensity, however, there is a significant increase in intensity of all three peaks P_1_, P_2_ and P_3_ in few-layer nanocomposites. While there are a number of theoretically-predicted phonon modes in the vicinity of these frequencies^46^, the specific identification of these peaks and assignment will require future detailed experimental studies which are beyond the scope of the current study. All our observations on Raman scattering are in line with recently reported studies in 2H-MoS_2_^43^,^44^,^45^. The change in line shape, intensity and peaks with decrease in number of layers suggest strong electron-phonon coupling and one can access the change in electronic properties with strains of these higher order peaks using this layer by layer process. While such studies are beyond the scope of the present paper which is the chromatic mechanical response in MoS_2_ based nanocomposites, nevertheless, it throws open new investigation into resonant Raman spectroscopy with strains. The resonant Raman scattering experiments confirm the structure is that of 2H-MoS_2_, differences in peaks with the number of layers in the nanocomposites, presence of van Hove singularities, and strong electron-phonon coupling, all of which can affect photomechanical actuation and energy applications.

**Figure 5:**
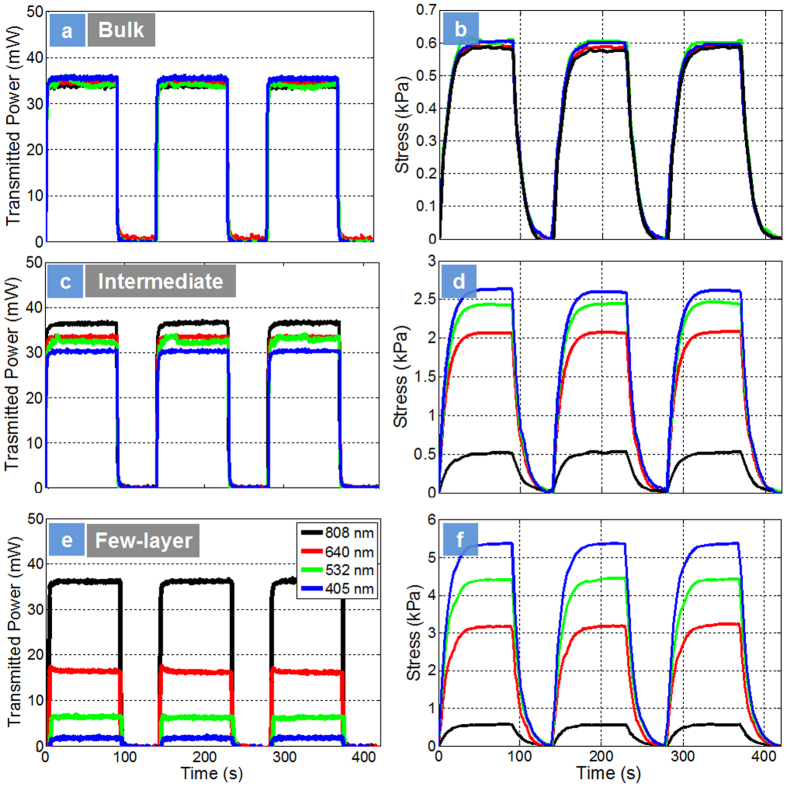
Photomechanical Actuation. Power transmitted through the sample and the corresponding photomechanical actuation of nanocomposites at different wavelength of light respectively: (**a,b**) bulk consisting of 100–500 layers; (**c**,**d**) intermediate consisting of 10–30 layers and (**e,f**) few layers consisting of 1–6 layers. The samples were all 0.1 wt.% nanocomposites using LBL process. No pre-strains were applied for these experiments.

Photomechanical responsivity testing on the nanocomposites is presented in Fig. 5. Figure 5(a) presents the transmitted power through the bulk nanocomposites and the corresponding photomechanical responsivity (Fig. 5b). For bulk nanocomposites, it is seen that power transmitted through the sample is similar irrespective of wavelength (Fig. 5a). They also exhibited similar photo-mechanical actuation stress at all wavelengths (Fig. 5b). A photomechanical stress value of ~0.6 kPa was measured. In these nanocomposites, the photo-mechanical actuation is due to the non-radiative decay of photons resulting in similar thermal response at all wavelengths. Figure 5(c) presents the power transmitted in the intermediate nanocomposites (10–30 layers) samples that suggest weak but selective absorption of light of different wavelengths. The corresponding photomechanical stress (Fig. 5d) is also dependent on the wavelength of light and ~4.5 times larger than bulk nanocomposites (Fig. 5(b)). The selective transmittance of light in these intermediate samples can be traced to the direct exciton peaks A and B indicated in magnified insert in Fig. 3(c). These two peaks were observed but weak in the intermediate samples. Correspondingly, the optical absorption and photomechanical response was also wavelength selective but the amplitude of mechanical response was small. Moving on to the few layer nanocomposites (Fig. 5(e)), the power transmitted through the sample is observed to be highly dependent on the wavelength of light. Again, this can be related to the strength of the direct exciton peak A and B in the nanocomposites in Fig. 3(c). It is seen that the most of the IR is transmitted (~72%) (1.53 eV) through the sample whereas most of the UV (97%) is absorbed (3.0613 eV). The results suggest a direct bandgap dependent absorption in the nanocomposite due to the unique structure of the MoS_2_ few layers. Figure 5(f) presents the photomechanical actuation of the few layer (1–6 layers) nanocomposites. One can observe not only wavelength dependence but extraordinary increase in mechanical stress as large as 10 times (~5.5 kPa) at 405 nm wavelengths (Fig. 5f). The photomechanical stress of few layer nanocomposites at 808 nm (below the bandgap) was also similar to bulk suggesting no significant optical absorption. The photoactuation stress at 640 nm and 532 nm follow the same trend as the optical absorption. The mechanical response is seen to be strong and follows the light absorption at 640 nm, 532 nm and 405 nm. The mechanical response is also reversible as when the light is switched off irrespective of the wavelength of light, the system comes back to its original state or reversible switching with one type of wavelength. To compare, in Azobenzene chromophores, reversible switching is accomplished with two different wavelengths (420 nm and 365 nm) between an extended *trans* and a shorter *cis* configuration^48^.

Scattering of light due to particle size larger than wavelength of light in bulk nanocomposites is a plausible explanation for decreased optical absorbance and wavelength insensitive photomechanical actuation. However, the small weight fractions of the MoS_2_ (0.1 wt. %) inside the optically transparent PDMS suggest most of the light may be transmitted through the sample rather than scattered. Further experiments were conducted to understand how optical absorbance, scattering and photomechanical response change with increase in weight fractions of bulk MoS_2_. Figure S3 (suppl.) presents the photomechanical actuation of bulk nanocomposites with increasing weight percentage of MoS_2_ additive. The transmitted power is also plotted on the same graph. An exponential decrease in transmittance is seen between 0.1 wt. % and 1 wt. % at all wavelengths. Above 1 wt. %, the power transmitted is almost zero suggesting strong light absorption. The rise in photomechanical actuation follows the same exact trend as the power absorbed by the sample. If the exponential decrease in transmittance in Figure S3 (suppl.) is as a result of dominant scattering with increase in weight fraction, this would result in significant decrease in photomechanical stress levels which is not observed. The insert in this Figure S3 presents the absorbance plot of the bulk nanocomposites with increase in weight fraction of MoS_2_ additive. The A/l is seen to follow the photomechanical actuation stress amplitudes. The extinction, absorbance and photomechanical stress amplitudes all increase with increase in MoS_2_ additive even for bulk over all the wavelength (300 nm to 1100 nm). Above 1 wt. % non-linear behavior is noted and saturation of optical absorption and photomechanical response. Thus we see two regimes in bulk nanocomposites. At low weight percentage of bulk MoS_2_ additive (~0.1 wt. %), high light transmission through the sample and geometric scattering cause lower photomechanical response. However, as the weight fractions of bulk MoS_2_ additive increases in the nanocomposite, optical absorption becomes dominant resulting in significant increase in photomechanical response. These results are also validated by the presence of van Hove singularity (peak L_2_, Fig. 4(a)) in the resonant Raman scattering experiments in bulk suggesting dominant absorption in these materials. It is interesting to note that, all the bulk samples irrespective of the weight fractions were insensitive to the wavelength of light suggesting the chromatic mechanical response is unique only to the few/intermediate-layer semiconducting structure of the 2H-MoS_2_ originating in the *d* orbitals. The direct exciton transition peaks A and B is the origin of the chromatic mechanical response and the strength of the A and B peaks determines the power transmitted and the overall mechanical response in few layer and intermediate nanocomposites. Thus, this is a unique photomechanical effect not seen in any other type of actuator. The origin of the photomechanical effect is as a result of optical absorption aided by the unique structure of the additive and its integration with the polymer chains. This also compares well with the past reports in this area of carbon nanotube and graphene based photomechanical actuators, where strong *sp*^2^ bonds of nanocarbons resulted in large amplitudes of photomechanical response^17^. In single layer graphene, all bonds are strong *sp*^*2*^covalent bonds that result in high intrinsic thermal conductivity, while reduced graphene oxide is a mixture of *sp*^2^ and *sp*^3^ bonds that resulted in smaller photomechanical actuation compared to pristine graphene. Finally, amorphous carbon and diamond-like carbon contain a large fraction of *sp*^3^ bonds that lower thermal conductivity and photomechanical actuation^17^. The large IR absorption in nanotubes is as a result of the van Hove singularity in the density of states. The optical absorption in graphene is dominated by the interband transitions. In 2H-MoS_2_ chromatic absorption is as a result of the *d* orbitals.

In MoS_2_, Ab initio calculations have suggested strong peaks in the visible region suggesting van Hove singularities in JDOS^19^. This leads to enhanced light absorption in TMDs, which is universal for all TMDs. The valence band consists of *4d* orbitals that are responsible for chromatic absorption, sending electrons from the ground state to the excited state in the conduction band depending on the energy of irradiated light. The strong covalent bonds (σ bonds) of the chalcogen atom couples this absorbed light due to the superposition of the *4d* orbitals of Mo and *3p* orbitals of S in the conduction band into an extraordinary thermal effect thus giving rise to this chromatic photomechanical effect. This is a unique mechanism not available to *sp* bonded materials such as carbon nanotubes and graphene, as well as plasmonic metallic gold nanoparticles.

**Figure 6:**
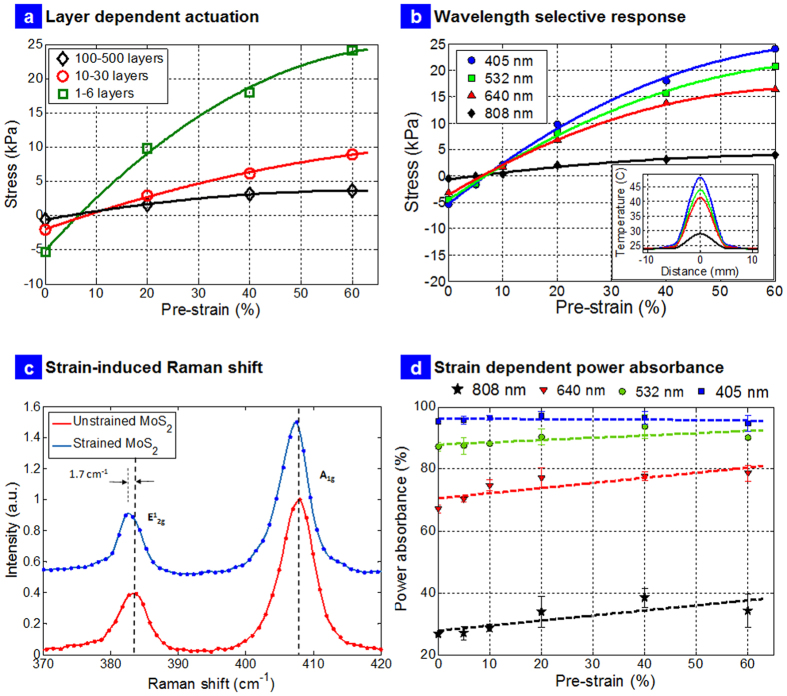
Photomechanical Actuation and Strain Engineering. (**a**) Magnitude of exerted stress as a function of pre-strains for different layered nanocomposites at 405 nm excitation; (**b**) Magnitude of exerted stress as a function of pre-strains at different wavelengths for the few-layer nanocomposite; insert is the actual temperature in the sample measured using thermocouples placed equidistant from center of the nanocomposite to the clamps; (**c**) Shift in the Raman vibrational modes E_2g_~ 1.7 cm^−1^ for strain of 10% for the few-layer nanocomposite; (**d**) Strain enhanced optical power absorption in few-layer nanocomposite.

Figure 6 presents the layer dependent actuation, wavelength selective responsivity, and mechanism of strain engineered optical absorption in these nanocomposites. Figure 6(a) shows the exerted actuation stress as a function of number of layers. It is observed that the number of layers affect the overall photomechanical response at all pre-strains with few layer nanocomposites showing the greatest change in photomechanical stress. Figure 6(b) presents the exerted stress as a function of pre-strains at different wavelengths. A stress of ~25 kPa is observed at 60% pre-strains for 0.1 wt. % few layer nanocomposite sample. This could potentially scale to 250 kPa/1 wt.% and significantly larger than graphene (50 kPa/2 wt.%)^12^ and could be useful both in micro and macroscopic actuation technologies based on light. The change in direction of photomechanical actuation (7–15% strain) is as a result of orientation effects and rubbery/entropic elasticity and has been observed in the past and is in line with previous reports on photomechanical actuators employing similar PDMS based nanocomposite system^9^. Temperature measurements were conducted with thermocouple placed equidistant from the center of the sample to the end of the clamps. The insert in Fig. 6(b) shows the Gaussian profile of the temperature given as.


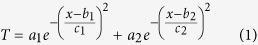


with the fitting parameters a_1_, b_1_, c_1_, a_2_, b_2_, and c_2_ that is dependent on the wavelength (Table S1, Suppl.). The temperature of the sample as a function of wavelength of the light was measured as: 48.1 °C (405 nm), 43.7 °C (532 nm), 41.1 °C (640 nm) and 29.0 °C (808 nm). Thus the lower thermal conductivity of TMD’s compared to graphene films cannot adequately explain these interesting results.

Local strain engineering and bandgap control has been reported in atomically thin samples of MoS_2_^27^,^32^. Tuning optical absorption with strains in TMDs seems like an interesting and exciting prospect for energy conversion in MoS_2_ due to its high strength and rapid reversal of bandgap. This is not possible using conventional thin film semiconducting materials which will undergo failure before any appreciable change in property can be produced^33^. Recent work has shown computationally that elastic strain is a viable agent for creating a continuously varying bandgap profile in an initially homogeneous, atomically thin layers but not experimentally explored completely^33^. In our few layer nanocomposites, applying global macroscopic uniaxial tensile strain of 10% using a custom made strain gadget (see Figure S4 suppl.) resulted in the shift of the E_2g_ mode by about−1.7 cm^−1^ and negligible change in A_1g_ as presented in Fig. 6(c). The change in Raman wavenumbers of−1.7 cm^−1^ suggests 1% strain transfer to the MoS_2_ from the matrix^27^. It has been suggested that the direct gap transition energy decreases for localized increasing uniaxial strain values for single layers; for a ~2.5% tensile strain, the reduction in direct bandgap transition energy of 5% for monolayers has been reported^27^. Since our sample is a nanocomposite consisting of layers of PDMS and MoS_2_, lower strain transfer was expected as the strains were applied globally to the composite as a whole and not locally engineered. Further, we also expected to see continuously varying strains in the MoS_2_ crystal due to the global application of the strains. These results are exciting in context of strain transfer into a macroscopic device achieving significant improvement in globalized photomechanical response which has not been reported yet.

Finally, another way to confirm that this is strain induced reduction in bandgap is to measure the power absorption directly at different strains (Fig. 6(d)). The results suggest 1.33 times increase in power absorption of ~808 nm photons at ~40% strains compared to unstrained value, which can only happen if the bandgap is reduced (average bandgap of ~1.71 eV was measured for few layers suspensions based on cutoff of the optical absorption is presented in Fig. 2(f)) and one has a continuously varying bandgap in the nanocomposite due to the stacked layers^33^. Similarly, 1.14 times change in power absorbed at 640 nm and 1.05 times change in power absorbed at 532 nm was observed at 40% strains. No significant change was observed in power absorption at 405 nm. These results suggest that as MoS_2_ layers in the nanocomposite were strained globally, a continuously varying strains developed inside the material resulting in more power being absorbed by the sample even at energies up to ~2.3 eV (higher than bandgap of single layers) that resulted in significantly better photomechanical response. It should be noted that although the design enables high strains to be applied to the sample, the strain transfer to the MoS_2_ additives inside the sample is between 1–5% for all the strains applied^27^, not enough to cause failure/delamination that can change the optical absorption irreversibly. Since our samples consisted of layers of MoS_2_ (1–6 layers) stacked on top of one another, stretching the sample globally must result in strain gradients and differential shift in the layers resulting in spatially varying energy levels of the stacked crystals that led to increase in optical absorption. Our results also suggest one can tune the power absorbed almost all wavelengths in the visible region in a macroscopic device with strains. Thus the strains, optical absorption and photomechanical response can be continuously, rapidly and reversibly tuned in these nanocomposites at different wavelengths and offers room for development of flexible energy harvesting devices in the future with advanced designs of our nanocomposites with scalability of localized strain engineering into macroscopic response. This does not happen in carbon nanotube^9^,^10^, graphene^11^,^12^ and gold nanoparticle based actuators^6^ and thus is a unique photomechanical effect due to the rich d electron physics in TMDs.

**Figure 7:**
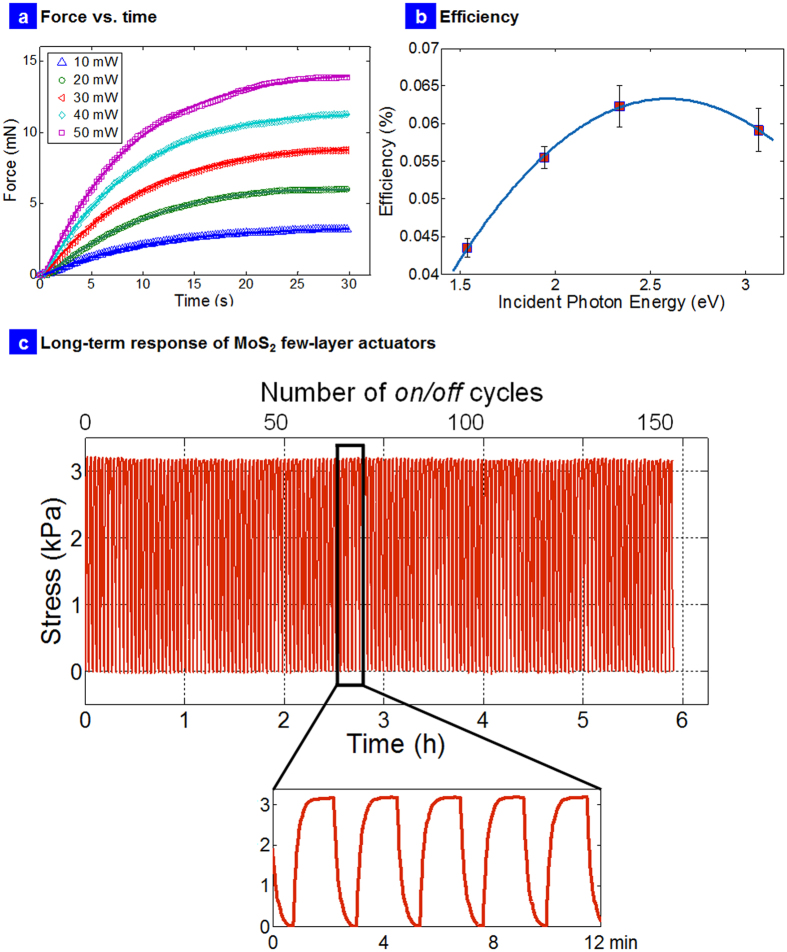
Force, Efficiency and Long term response. (**a**) Force versus time at different laser power; (**b**) Efficiency versus incident photon energy; (**c**) Long term response of the 2H-MoS_2_ few layer actuators operated over ~6 hours (~150 cycles) at 640 nm wavelength suggesting excellent stability.

Figure 7 presents some figures of merit for the few layer nanocomposite actuators. A photo actuation force of 10 to 15 mN at different laser power (405 nm) is observed (Fig. 7a). Force as a function of time at different laser power suggests wavelength and intensity dependent actuation similar to Azobenzenes^3^. An important aspect of any actuator is energy efficiency at converting light of different illumination into mechanical movement. Therefore, the efficiency (η) of the MoS_2_ composites to a known illumination source was evaluated^17^. Figure 7(b) presents the efficiency relationship as a function of incident photon energy for few layer nanocomposites. A significant increase in efficiency was noted at wavelengths between 808 nm to 405 nm. The highest efficiency achieved was ~0.06% at ~2.5 eV followed by a small drop at higher energies. The efficiencies were calculated exactly as in our previous report^17^. The increased thermal effect at higher energies decreased the efficiency slightly. However, these numbers are still impressive for photo generated work in polymers. In comparison, past–light driven actuators based on polyvinyledene difluoride (PVDF) have demonstrated an efficiency value of ~8.3 × 10^−5^% under quasi-static motion, about three orders of magnitude less efficient than our exfoliated MoS_2_-elastomer systems (~6 × 10^−2^%). Finally, the efficiency was twice as high as recently reported graphene-elastomer photo-mechanical actuators (0.03%)^17^ with other excellent features such as wavelength selective response and could find applications in nanopositioning^17^, plastic motors^18^ and micro-opto-mechanical systems^14^ (MOMS).

Figure 7(c) presents the long term stability of the few-layer photomechanical actuator using 640 nm laser excitation over 150 cycles or 6 hours of continuous operation. No pre-strains were applied to the sample. The slightly greater than ~3 kPa matches exactly the photomechanical actuation experiments presented in Fig. 5(f) at 640 nm wavelength (the time delay between these experiments is ~3–6 months). It should also be noted that the long term experiments in Fig. 7(c) were conducted after the strain based optical absorption experiments presented in Fig. 6(d). There are several conclusions that can be deduced from these long term experiments. First, the photomechanical actuation of the 2H-MoS_2_ few-layer nanocomposite is fully reversible and robust actuation mechanism. Second, the strain based optical absorption experiments did not delaminate the interface/sample, which would otherwise cause an irreversible change in the amplitude of photomechanical actuation stress (a significant reduction in stress) due to increased scattering in these long term experiments. Third, the strain gradients on the MoS_2_ crystals and the change in bandgap is also completely reversible as the sample exhibits pristine-like behavior (0% strain behavior) with the same amplitude of mechanical response in these long term experiments. The strain transfer to the MoS_2_ additive is thus in line with previous reports^27^ and should be in between 1–5% in our samples at the highest strain applied to the nanocomposite, not enough to cause failure, delamination and degradation all of which will irreversibly change the long term photomechanical response. This also lends credence to the high strength of the few layer MoS_2_ additive. Thus the high strength and strong light-matter interactions in 2H-MoS_2_ giving rise to this robust chromatic mechanical response is not only a new mechanism but also a new material design for future macroscopic photo mobile polymer networks based on TMDs.

## Methods

### Liquid Phase Exfoliation, Imaging and Optical Absorbance Spectroscopy

The bulk MoS_2_ solution was obtained by 7-hour bath sonication of MoS_2_ Ultrafine Powder obtained from Graphene Supermarket. The intermediate solutions were sonicated for 50 hours, and then centrifuged at 1500 rpm for different time durations (45 to 120 minutes). The few layer samples were bought from Graphene Supermarket in ethanol and used as such. The number of layers were characterized for each type of suspension using AFM. The AFM images were taken using a NaioAFM of Nanosurf in tapping mode with a cantilever resonance frequency of ~148 kHz. HRTEM was conducted using an FEI Tecnai transmission microscope (TM) (model G2 F20). FFT was obtained from the HRTEM image. The operating voltage was set at 200 kV. The sample was prepared by dropping one drop of exfoliated MoS_2_ in ethanol solution on lacey-carbon Cu grids (Ted Pella Inc., CA). The Cu grids were dried in air to make sure there are sufficient isolated flakes to be observed. SEM images were obtained using a JEOL JSM-7000F instrument at 20 kV of power and under an ultra-high vacuum, 10^−5^ Pa. Secondary electron detector was utilized at 8 mm working distance to capture high-resolution images of MoS_2_ flakes at magnifications as high as 100,000X. The optical absorbance of the MoS_2_ solutions and the nanocomposites were measured using a Hitachi U–5100 ratio beam spectrophotometer in a wavelength range of 300 nm-1100 nm.

### Sample Preparation

#### Bulk MoS_2_ actuators

10.0 mg as-received ultrafine nanopowder from Graphene Supermarket is weighed and added to 100 mL of ethanol (200 proof Sigma Aldrich), and sonicated for 7 hours in a bath sonicator. Using this solution, 2 mL of MoS_2_ in ethanol is then used (200 μg MoS_2_) for vacuum filtration process resulting in a mass per area of MoS_2_ nanoparticles of 0.18601 g/m^2^ on filter membrane (Whatman Anodisc). Area is calculated based on the diameter of the deposited film. Here 47 mm diameter Anodisc membrane after vacuum filtration resulted in 37 mm diameter of deposited mass. This resulted in 0.18601 g/m^2^. This layer is then transferred to an 80 μm thick PDMS layer and then another layer of 80 μm thick PDMS is spun coated on top of this layer. The mass of the two layers of PDMS is then measured by cutting known areas of the neat PDMS (i.e. the clear part without any MoS_2_) and calculating the mass/area of PDMS layers. This process is repeated several times for each sample to get averages. The ratio of the mass per area of the MoS_2_ to that of the mass per area of the PDMS gives the weight percentage of the MoS_2_ in the composite samples (i.e. 0.1 wt. %).

### Intermediate MoS_2_ actuators

The process for intermediate samples is the same as bulk except that we used 50 hours sonication and then the solution is centrifuged at 1500 rpm. The top and bottom parts of the solution is then separated similar to reported in literature^39^. The concentration of the centrifuged solution is then calculated by weighing the amount of MoS_2_ nanoparticles removed from the top parts of each vial and depositing it on filter membranes. This process is repeated for several samples to ensure repeatability. The concentration of the used centrifuged solution for the intermediate nanocomposites is also verified using the Lambert-Beer law and optical absorbance method^39^. Using 11.628 mL of the centrifuged solution with a concentration of 0.0172 mg/mL resulted in 200 μg of MoS_2_ in the solution. This 11.628 ml sample is then vacuum filtered on a filter membrane (deposited material diameter of 37 mm) arriving at a mass per area concentration of 0.18601 g/m^2^. The same process as presented above in bulk is accomplished to reach a 0.1 wt. % of intermediate nanocomposites.

### Few-layer MoS_2_ actuators

As-received MoS_2_ solution (Graphene Supermarket) is used in the fabrication process. Because of ultra-small size of few-layer nanoparticles, transferring to PDMS layer cannot be accomplished from Anodisc membrane and is done using an evaporative drop deposition method. The initial concentration of the purchased few-layer solution as per manufacturer specification was 18 mg/L. 258 μL of this solution (4.65 μg of MoS_2_) is then deposited directly on the 5 mm × 5 mm sample area of the PDMS to reach the same mass per area as bulk and intermediate samples (i.e. 0.18601 g/m^2^). The final weight percentage (0.1 wt.%) calculation is the same as the bulk and intermediate samples after the second PDMS layer is spun coated on top of the PDMS/MoS_2_ layers.

### Stress Test Experiments

MoS_2_/PDMS actuators were fabricated based on a layer-by-layer (LBL) method. PDMS silicone elastomer obtained from Dow Corning (Sylgard 184) was used as the host matrix. PDMS is a two part solvent-free flexible silicone organic polymer in the form of a base compound with a separate hydrosilane curing agent that acts as a cross-linker. The term cross-linking ratio (CLR) used throughout the paper refers to the ratio of PDMS cross-linker to the base compound. The PDMS base was mixed in 1:10 ratio to the cross-linker and then deposited on a glass slide. A standard spin coating process at 750 rpm for 90 seconds produces 80 μm thick film of PDMS on the glass slides. The films are then cured at 120 °C for 20 minutes and post-cured for 12 hours at room temperature. A uniformly distributed layer of MoS_2_ nanoparticles deposited on Whatman Anodisc inorganic membrane using a standard vacuum filtration process is then transferred into the first layer of PDMS. Another layer of PDMS is then spin coated and cured on top of the MoS_2_ layer resulting in a layer-by-layer structure of about 160 μm thick. The samples are then cut in 50 mm × 5 mm strips (30 mm of active test area with 10 mm on each side for fixture mounting). All experiments were conducted in a climate-controlled laboratory. The photomechanical testing system, strain application, lasers all form an automated system that is placed inside a black box to avoid stray light from interfering the experiments. The laser sources include wavelengths from ultraviolet to near-infrared regions (405 nm, 564 nm, 640 nm, and 808 nm) all with the same laser power of 50 mW and beam diameters of ~3 mm. The laser diode was placed ~100 mm from the middle of the test strip such that the illumination impacted normal to the PDMS/MoS_2_ surface. More detailed information on photomechanical stress tests are available^12^. Long term stress test experiments for stability and robustness of the actuation mechanism was conducted continuously over 6 hour period.

#### Strain Engineering using Raman Spectroscopy

A custom strain gadget was developed using design and laser cutting clamps. The stretching fixture was developed with 500 μm resolution and 5 mm initial clamps’ spacing. This fixture was used to study the effect of strain on the Raman shifts of polymer/MoS_2_ nanocomposite actuators with 10% increments shown in Figure S4. The Raman measurements were performed by the excitation laser line of 532 nm using a Horiba XploRA Raman system in ambient air environment. The power of the excitation laser line was kept at 1 mW. Since there was two 80 micron PDMS layers in between the MoS_2_ layer, the incident optical power was significantly less than 1 mW, not enough to induce heating effects in line with previous reports^44^. The laser beam was focused onto the surface of the samples using a 100× objective lens. The measurements were repeated for at least 5 different points on each sample.

### Resonant Raman Scattering

Microscopic measurements of the nanocomposite optical spectra were performed in backscattering geometry with a resonant excitation. The 632 nm line of a He-Ne laser was used for these measurements. All measurements were done using an optical filter with an incident power of ~1 mW. Since there was an 80 μm PDMS layer on top, the incident power is significantly less than 1 mW, not enough to induce local heating effect in line with previous reports on resonant Raman scattering^44^. The size of the laser spot on the sample was ~1 μm. A Leica microscope objective (50 X) was used to excite the sample and collect the emitted light. The collected spectra were dispersed by a Renishaw inVia Raman microscope system, equipped with a multi-channel high resolution Si-CCD device. The spectrum of pure PDMS was measured and subtracted from the measurements taken from bulk, intermediate and few layer nanocomposites. The broadened peaks were deconvoluted to show the overlapping components.

## Additional Information

**How to cite this article**: Rahneshin, V. *et al*. Chromatic Mechanical Response in 2-D Layered Transition Metal Dichalcogenide (TMDs) based Nanocomposites. *Sci. Rep.* **6**, 34831; doi: 10.1038/srep34831 (2016).

## Supplementary Material

Supplementary Information
