# Supplementary material for: Chromatic Mechanical Response in 2-D Layered Transition Metal Dichalcogenide (TMDs) based Nanocomposites
Source: Sci Rep. 2016 Oct 7;6:34831. doi: 10.1038/srep34831 (PMC5054383; doi:10.1038/srep34831)
Supplement: Supplementary Information [file srep34831-s1.pdf]

Chromatic Mechanical Response in 2-D Layered Transition Metal Dichalcogenide (TMDs) based Nanocomposites

Vahid Rahneshin[1], *Farhad Khosravi*[1], Dominika A. Ziolkowska[2,3], Jacek B. Jasinski[2] *and Balaji Panchapakesan*[1]*

[1]*Small Systems Laboratory*

Department of Mechanical Engineering

Worcester Polytechnic Institute, Worcester, MA 01609

[2]*Conn Center for Renewable Energy Research*

University of Louisville, Louisville, KY 40292

[3]*Faculty of Physics, University of Warsaw*

Pasteura 5, 02-093 Warsaw, Poland

**bpanchapakesan@wpi.edu*

Table of Contents

Figure S1: AFM imaging of number of layers 2

Figure S2: Cross-sectional SEM of 0.1 wt. % bulk nanocomposites 3

Figure S3: Photoactuation of bulk nanocomposites 4

Table S1. The Gaussian's parameters 5

Figure S4: Strain engineering device 6

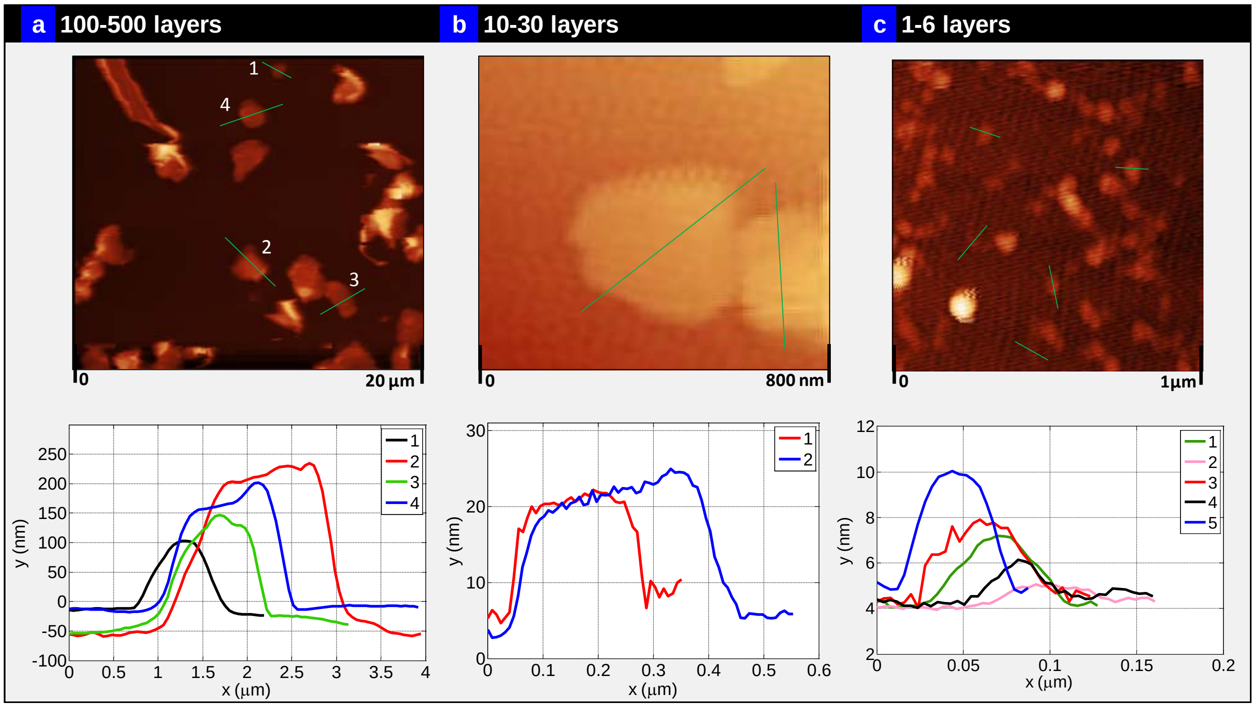

Fig S$_1$. Atomic force microscopy images and Z axis height measurements of the flakes

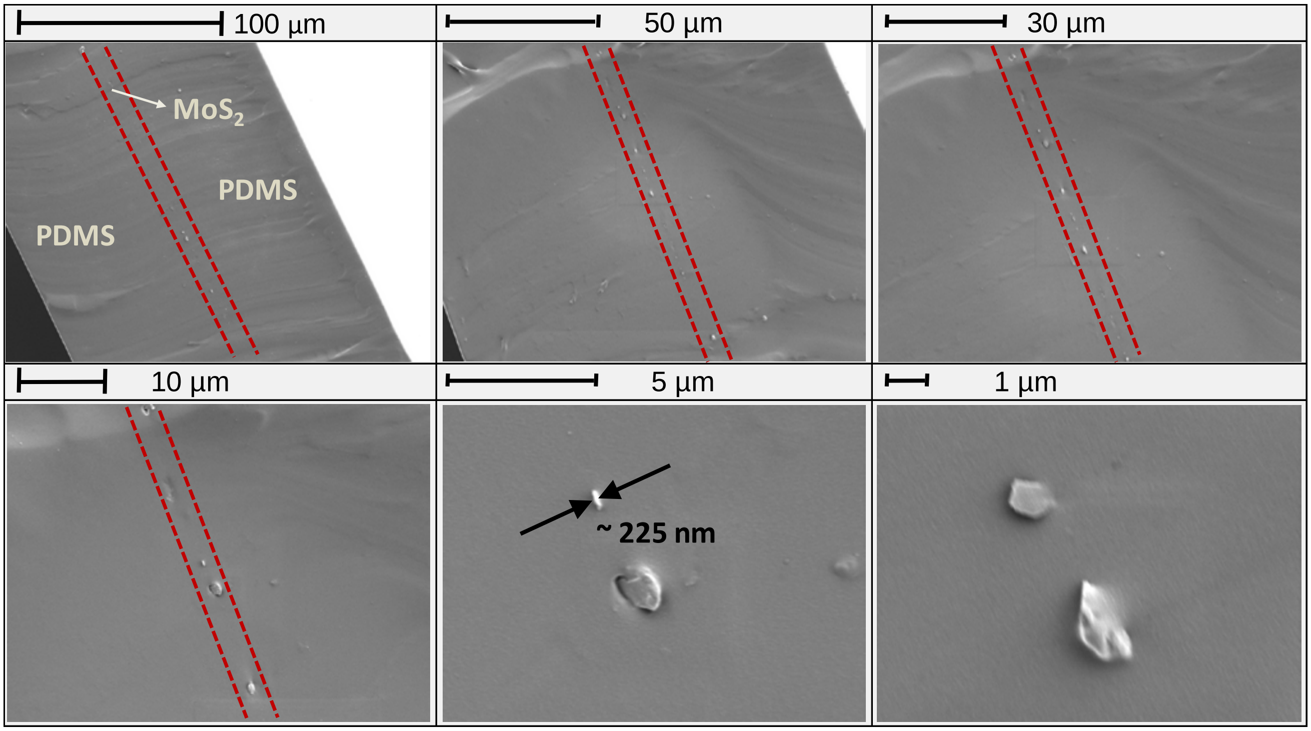

Fig.S$_2$. – Cross-sectional SEM of 0.1 wt. % bulk nanocomposites

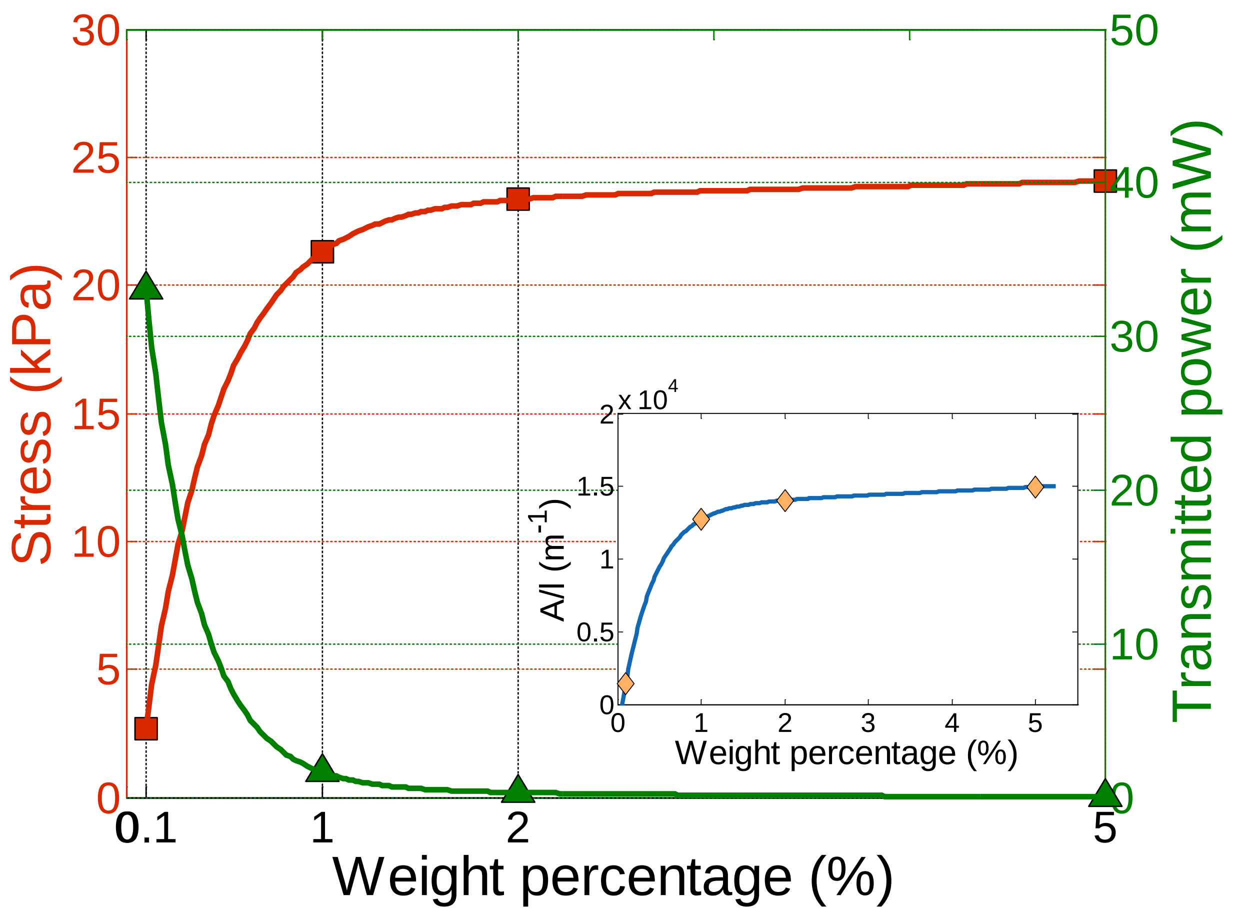

Fig.S3: Photo-actuation of bulk nanocomposites with different weight percentage at 40% pre-strain. The transmitted power is also plotted. The input power in all experiments is fixed at 50 mW, 640 nm wavelength. The insert is the UV-Visible spectroscopy at 670 nm as a function of weight fractions of bulk nanocomposite.

Table S1. The Gaussian's parameters of wavelength selective temperature in the few-layer samples

	a_1	b_1	c_1	a_2	b_2	c_2
**405 nm**	24.59	-0.002067	3.176	28.45	2384	5549
**532 nm**	20.34	-0.001033	3.152	25.61	2593	8925
**640 nm**	17.59	-0.01081	3.104	2038	1489	7057
**808 nm**	5.238	-0.00836	3.186	27.49	2203	5776

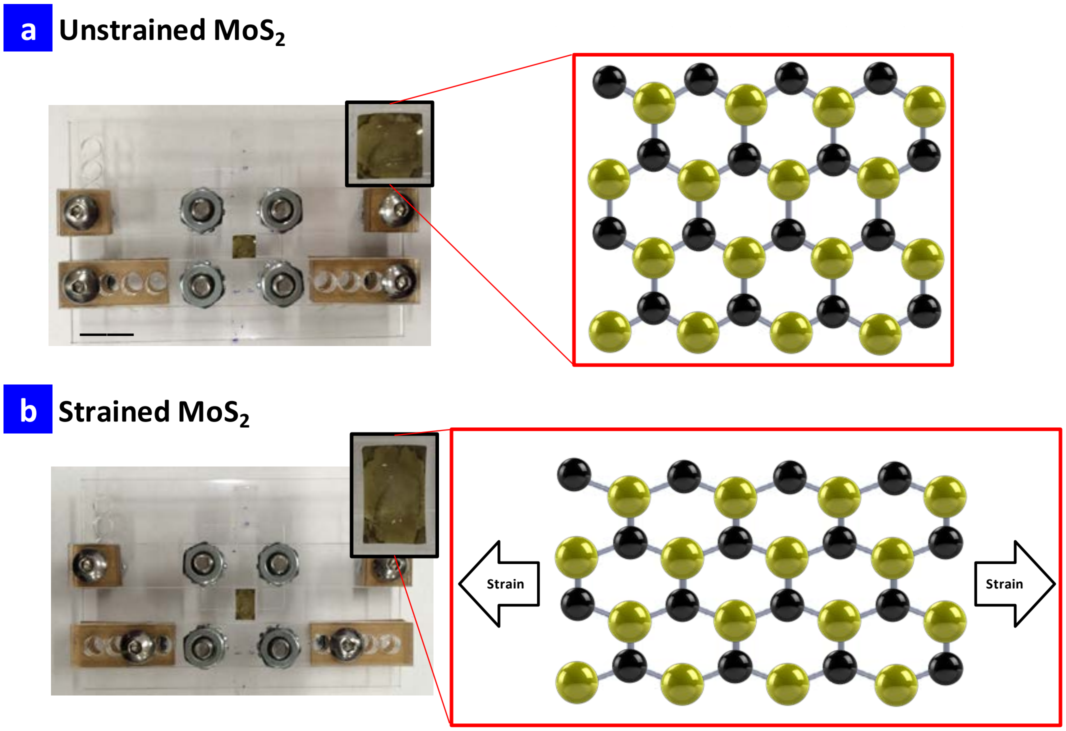

Fig S4. Strain Engineering Device.
